# Spray-dried porcine plasma improves piglets’ performance and modulates gut immune-related genes in the first week post-weaning

**DOI:** 10.5713/ab.25.0185

**Published:** 2025-06-10

**Authors:** Zacharia Waithaka Ng’ang’a, Nuria Tous, Muzahir Hussain, Maria Ballester, Javier Polo, Raúl Beltrán-Debón, Joan Tarradas, David Torrallardona

**Affiliations:** 1 Animal Nutrition Program, Institute of Agrifood Research and Technology (IRTA), Constantí, Spain; 2 MoBioFood Research Group, Department of Biochemistry and Biotechnology, Universitat Rovira i Virgili, Tarragona, Spain; 3 Animal Breeding and Genetics Programme, Institut de Recerca i Tecnologia Agroalimentàries (IRTA), Torre Marimon, Caldes de Montbui, Spain; 4 APC Europe, Granollers, Spain

**Keywords:** Immune Response, Intestinal Health, Spray-dried Porcine Plasma, Weaned Piglets

## Abstract

**Objective:**

This study aimed to explore the effects of spray-dried porcine plasma (SDPP) in pre-starter feed of piglets on performance, fecal consistency, gut integrity biomarkers, and gene expression patterns related to intestinal function.

**Methods:**

Ninety piglets (18 pens; 5 pigs/pen; 8.16±1.29 kg initial body weight) were offered pre-starter feed with either soy protein concentrate (control) or SDPP for 14 d post-weaning, and a common commercial starter feed between 14 and 35 d. Pigs’ performance was recorded at 0, 7, 14 and 35 d of trial and their fecal consistency was assessed on the first 2 weeks. At 7 d, one piglet per pen (18 in total) was sampled for blood, intestinal mucosa, and bile. Blood serum calprotectin and citrulline and bile sIgA were quantified with ELISA, and gene expression in the mucosa from ileum, jejunum, and caecum was analyzed with high-throughput microfluidic technology.

**Results:**

Relative to control, SDPP improved feed intake (215 vs 280 g/d; p<0.05), weight gain (133 vs. 205 g/d; p<0.05) and gain-to-feed ratio (0.61 vs. 0.74; p<0.05) between d 0 to 7. Serum calprotectin and citrulline, and bile sIgA at d 7 were not affected. Piglets fed SDPP had higher expressions of *IL-1β* (p = 0.033) in jejunal mucosa, and of *IL-1β* (p = 0.018), *IL-8/CXCL8* (p = 0.010), *GBP1* (p = 0.014) and *TGF-β1* (p = 0.015) in ileal mucosa, at 7 d post-weaning. No effects on fecal scores were observed.

**Conclusion:**

It is concluded that the administration of SDPP during the pre-starter phase improves piglet’s performance during the first week post-weaning. These SDPP associated benefits appear to have been achieved through the modulation of gut homeostasis, potentially involving the regulation of inflammatory factors in the small intestinal mucosa.

## INTRODUCTION

Weaning is a critical phase in pig production that imposes significant stress on piglets, leading to reduced feed intake, impaired growth, and compromised gut barrier function, thus increasing the risk of intestinal inflammation, morbidity, and mortality, particularly during the first week post-weaning [1,2]. There has been a rising interest in innovative nutritional strategies that can effectively support the health and performance of weaned piglets in the absence of antibiotics and zinc oxide [3]. Spray-dried porcine plasma (SDPP) has been postulated as a gut health promoting ingredient and has been included into piglet diets at levels of up to 6% [4,5]. As a protein source proposed for use in pig diets in the late 1980s [6], SDPP has been shown to improve piglets’ performance, intestinal barrier function, immune responses and to reduce the incidence of post-weaning diarrhea [7–9]. These benefits of SDPP have been attributed to the presence of biologically active compounds such as growth factors, cytokines and immunoglobulins (importantly IgG) [8,10]. Some studies have observed that SDPP influences the intestinal microbiota composition [11], prevents the binding of pathogens to the gut epithelium [10], reduces intestinal inflammation [1], improves intestinal morphology [9], and modulates immune cell activity [12], which collectively support gut homeostasis and performance in weaned piglets.

Whereas reviewing nutrition and gut health of the young pig around weaning, Lallès [13] concluded that spray-dried plasma (SDP) represents one of the most satisfactory alternative solutions capable of limiting gut problems post-weaning in the absence of preventive use of antibiotics. Hedegaard et al [14] also gave both *in vitro* and *in vivo* evidence to support the idea that natural IgG directly purified from pig plasma and given as a feed supplement can be used in modern swine production as an efficient and cost-effective means for reducing both the occurrence of post-weaning diarrhea and the usage of antibiotics. These authors also suggest that SDPP may have potential for the prevention and treatment of other intestinal infectious diseases caused by unknown agents. Although substantial literature supports these benefits and current causal understanding suggests that SDPP improves growth rates by increasing feed intake and offering barrier integrity protection, the underlying molecular mechanisms particularly those related to intestinal gene expression remain poorly understood. Most of the previous research on SDPP and other alternatives has focused either on performance or post-weaning diarrhea, leaving a significant knowledge gap in terms of how SDPP modulates gut physiology and immune function at the molecular level.

This study aims to address this gap by investigating how SDPP influences performance, gut health biomarkers and gene expression in the small intestine of piglets during the critical post-weaning phase, ultimately to provide a clearer comprehension of its functional mechanism of action. We hypothesized that SDPP enhances early post-weaning performance and enhances gut health by modulating the gene expression patterns associated with gut barrier function, digestive enzymes and hormones, immune response, stress response, and nutrient transport during the immediate post-weaning period. To explore this hypothesis, we applied high-throughput quantitative real-time polymerase chain reaction (qPCR) based on microfluidic chips [15], to assess the expression of 45 targeted genes in the jejunal and ileal mucosa, complemented by performance and gut health indicators to provide a comprehensive evaluation of SDPP’s impact in the early post-weaning period. Part of the results have been preliminarily published in abstract form [16].

## MATERIALS AND METHODS

### Piglets’ housing and management

**Figure 1:**
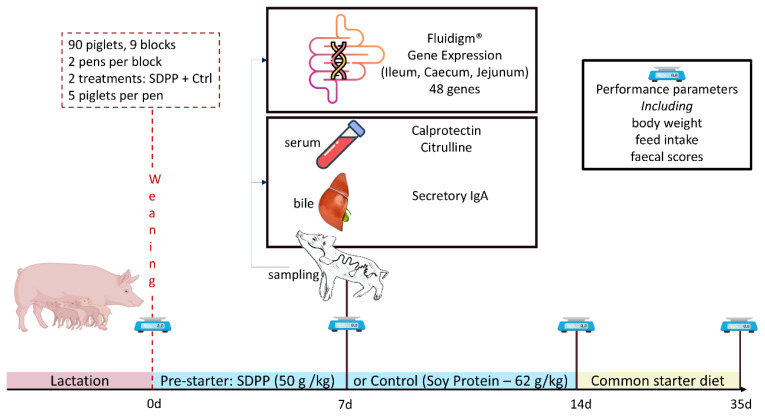
Schematic illustration of the study design, sampling and lab analyses. SDPP, spray-dried porcine plasma.

The study (illustrated in Figure 1) was conducted in the post-weaning facilities of the experimental farm of IRTA’s Animal Nutrition Programme (Centre Mas Bové). A total of ninety newly weaned piglets ([Large White×Landrace]×Pietrain; mixed sexes (total of 41 barrows and 49 gilts); 8.16±1.29 kg body weight [BW]) of around 26 d of age, obtained from IRTA’s sow herd, were used. The piglets were housed in two weaning rooms with 12 and 6 pens, respectively (1.82 m^2^; five piglets per pen). Each room was provided with automatic heating, forced ventilation and completely slatted floors. Feed was distributed *ad libitum* in one feeding trough per pen with four feeding spaces.

### Experimental diets

Piglets were offered pre-starter (PS) diet specifications between 0–14 d in mash form, and starter (ST) diet specifications between 14–35 d in pelleted form. There were two experimental treatments that were offered between 0–14 d with the PS diet specifications: a control diet with soy protein concentrate (Soycomil P; Andreu Pintaluba) at 6.2% and a test diet with SDPP (Appetein, APC Europe) at 5.0%. Feeds were formulated to be isonutritive and isoproteic and to meet or exceed the minimum nutrient requirements as recommended by FEDNA [17]. The composition of the diets is presented in Table 1. The gross energy of feeds were determined by NIR spectrophotometry, using a prediction model that fulfil all the quality requirements stated in ISO 12099:2017 “Animal feeding stuffs, cereals and milled cereal products — Guidelines for the application of near infrared spectrometry”, as follows (Parameter & Reference method AOAC 2000 [Prediction model R^2^ / Standard Error of Prediction / Uncertainty (±2×SEP)]: Gross energy (kcal/kg) DIN 51900 [0.94/33/66]. Dry matter, crude protein, total fat, and ash were determined by the AOAC methods 925.09, 968.06, 920.39, and 942.05, respectively. Feed and water were provided *ad libitum* to all the animals throughout the whole experimental period.

### Experimental design and sampling

Piglets were distributed and treatments were assigned according to a randomized complete block design. At the start of the trial, a total of 90 piglets were selected and randomly distributed by initial BW into nine blocks (sex was not considered as a blocking criteria). Each block consisted of two pens with five piglets each. Within each block, the two dietary treatments were randomly distributed among the two pens.

At d 7 post-weaning one animal per pen (the animal closest to the pen’s median initial BW; 18 animals in total) was sampled for blood and euthanized for the sampling of intestinal mucosa and bile. Blood serum and bile samples were collected in cryotubes, frozen immediately in dry ice and kept at −80°C. Fragments of 10 cm of jejunum (at 600 cm proximal from ileo-cecal valve), ileum (at 50 cm proximal from ileo-cecal valve) and cecum (at 5 cm distal from ileo-cecal valve) mucosa were obtained and after cleaning with saline solution they were scrapped, collected in 2 mL cryotubes and stored at −80°C.

### Growth performance and fecal score

Feeder and piglets were weighed at weaning (d 0), at d 7 and 14, and at the end of the experiment (d 35). Initial and final BW, average daily weight gain (ADG), average daily feed intake (ADFI) and gain-to-feed ratio (G:F) were calculated for each pen. Fecal consistency was assessed daily based on direct observation of individual piglets in each pen during the first and second weeks of trial, using a 5-category score system (0 = firm and shaped; 1 = soft and shaped; 2 = soft without shape; 3 = loose; 4 = watery) modified from the 4-category fecal consistency classification scale by Pedersen and Toft [18].

### Gut barrier and immune indicators analyses

Commercial ELISA kits were utilized for the measurement of the concentrations of serum calprotectin and citrulline, and bile sIgA according to the manufacturer’s instructions (CSB-EQ013485PI, Cusabio; K 6600, Immundiagnostik AG; and abx258080, Abbexa, respectively). Absorbance was measured at 450 nm using a microplate reader (Biotek Epoch, Biotek Instruments), and the concentrations for each marker were determined by comparing the optical density of the samples to a standard curve.

### RNA extraction and reverse transcription

Total RNA was extracted from the jejunal, ileal, and cecal mucosa of individual pigs using the miRNeasy Mini Kit (Qiagen) following the manufacturer’s instructions. The RNA concentration and purity were determined using NanoDrop ND-1000 spectrophotometer (Thermo Fisher Scientific), and the extracts were stored at −80°C until further use. Complementary DNA (cDNA) was synthesized from one μg of each RNA sample using the PrimeScript RT Reagent kit (Takara, RR037A; Dalian), according to the manufacturer’s protocol.

### Pre-amplification

Pooled cDNA samples were used to generate a standard curve for quantification during qPCR. All cDNA samples, diluted 1:5 with DNA suspension buffer (10 mM Tris-HCl, 1 mM EDTA, pH 8.0), were pre-amplified using a Preamp Master Mix (Standard Biotools) combined with a custom primer mix containing all 48 primer pairs (500 nM each) for subsequent qPCR analysis. The pre-amplification thermocycling conditions were as follows: initial denaturation at 95°C for 10 min, followed by 15 cycles at 95°C for 15 s and 60°C for 4 min. After pre-amplification, the reactions were cleaned up using Exonuclease I (Exo I) to remove residual primers. The pre-amplified cDNA -Exo I treated samples were then diluted 1:20 with DNA suspension buffer prior to analysis.

### High-throughput gene expression: quantitative real-time polymerase chain reaction analysis

High-throughput qPCR was performed using the BioMark X9 system (Standard BioTools, formerly Fluidigm) with 96.96 Dynamic Array Integrated Fluidic Circuit (IFC) chips, allowing the combination of 48 primer pairs in duplicates with 96 samples. qPCR primer sequences used in this study consisted of both in-house designs and modifications of some primers employed by González-Solé et al [19], purchased from Condalab, and are listed in Supplement 1. Genes below the detection limits in most samples were replaced and excluded from further analysis.

The qPCR sample mix was prepared by combining 2.7 μL of pre-amplified and Exo-I treated samples (diluted 1:20 with DNA suspension buffer), 3.3 μL of 20× EvaGreen DNA Binding Dye (Biotium, PN 31000), and 0.5 μL of 20× DNA Binding Dye Sample Loading Reagent (Fluidigm, PN 100–0388). For each assay, the mix consisted of 30 μL of 2X Assay Loading Reagent, 27 μL of DNA suspension buffer and 3 μL of forward and reverse primers, each at a final concentration of 5 μM.

A volume of 5 μL of each sample mix and 5 μL of each assay mix were loaded into the respective inlets on the 96.96 Dynamic Array IFC chips. Non-template controls were included in each chip to detect any contamination or nonspecific amplification. Standard curve dilutions, prepared from pooled cDNA samples, were also added to the chip to enable accurate quantification.

The chips were then transferred to the BioMark X9 system for thermal cycling and fluorescence detection. The thermal cycling conditions were as follows: an initial activation step at 95°C for 60 s, followed by 35 cycles at 96°C for 5 s and 60°C for 20 s. Data acquisition was performed at the end of each cycle, and a melting curve analysis was conducted to confirm specific amplification.

BioMark system Real-Time PCR Analysis software (version 1.0.2; Standard BioTools) was used for initial quality control and threshold setting. Further data processing and normalization were conducted using the DAG expression software (www.dagexpression.com) [20], with corrections for PCR efficiency for each primer assay. Four porcine intestinal reference genes (*GAPDH*, *ACTB*, *HPRT1*, and *TBP*) were considered in this study. Based on stability analyses using DAG software, *TBP* and *GAPDH* were identified as the most stable and were used as reference genes for normalization.

### Statistical analysis

Performance variables were analyzed using the proc MIXED model procedure in SAS software (SAS 9.4; SAS Institute). The model included blocks of initial BW as a random effect and treatment as the fixed effect. The pen was considered as the experimental unit. Results are expressed as least square means. Fecal consistency assessed individually was analyzed using a Chi-square test of independence utilizing the PROC FREQ procedure in SAS software. The graphical illustration of fecal score frequencies was generated using the GraphPad Prism software (version 8.0; GraphPad Software). Differential relative gene expression and ELISA biomarkers in ileum, jejunum, and cecum were analyzed using the GLIMMIX procedure in SAS software. A mixed-effects model was employed, with treatment as a fixed effect and block as a random effect. Results are expressed as least square means. Statistical significance was determined at a p-value of less than 0.05 and between 0.05 and 0.10 was considered as tendency.

## RESULTS

**Figure 2:**
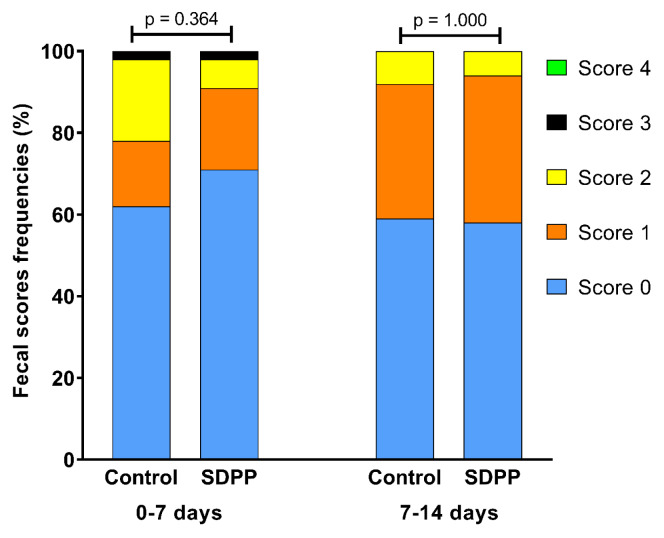
Fecal score frequencies of individual piglets based on daily direct observations between 0–7 and 7–14 d of experiment calculated using the maximum score given to each piglet within each period. SDPP, spray-dried porcine plasma.

The performance results considering average pen values are shown in Table 2. During the first week of the PS phase (0–7 d), piglets that were offered SDPP had higher ADFI (280 g vs 215 g, p<0.05), ADG (205.3 g vs 132.8 g, p<0.05) and G:F (0.74 vs 0.61, p<0.05) than those on the control diet. No differences between control and SDPP treatments were observed during the second week of the PS phase (7–14 d) nor during the ST phase in which the piglets were offered a common ST diet (14–35 d). No differences in fecal score frequencies between treatments were observed on the first and second weeks of experiment (Figure 2). The Calprotectin and Citrulline concentrations in serum were not different between treatment groups, nor that of sIgA in bile (Table 3).

The gene expression profiles for key genes involved in the overall gut function, grouped under functional groups including metabolic, enzymatic, and hormonal activity, immune response, nutrient transport, stress indicators, and barrier function, studied relative to the reference genes in the different intestinal sections are presented in Table 4. Noticeably, differences were only observed for four genes that play roles in inflammatory processes which were upregulated by SDPP. The expression of Interleukin 1 beta (*IL-1β*) increased with SDPP inclusion in both the jejunum (p<0.05) and ileum (p<0.05). In the ileum, the SDPP-fed piglets also showed higher expression of C-X-C motif chemokine ligand 8 (*CXCL8*, commonly referred to as *IL-8*) (p<0.05), Guanylate binding protein 1 (*GBP1*, p<0.05) and Transforming growth factor beta 1 (*TGF-β1*, p<0.05) genes. The expression of Inhibitor of nuclear factor kappa B kinase subunit beta (*IKBKB*), Toll-like receptor 2 (*TLR2*), Nuclear factor kappa B subunit 1 (*NFKB1*) and Intestinal alkaline phosphatase (*ALPI*) tended to be increased in the ileum of the SDPP group compared to the control group (p<0.1). In cecum, only Interleukin 10 (*IL10*) showed a tendency to be downregulated by SDPP (p = 0.068).

## DISCUSSION

SDPP is commonly included in PS pig diets for its positive effects on growth performance, which were initially attributed to its palatability, which enhances feed intake during the critical post-weaning period [9,21]. Additionally, SDPP has been associated with health benefits, particularly due to the presence of bioactive compounds that may play an important role in modulating immune responses and strengthening intestinal barrier function [7,12,22]. In this study, we investigated the effects of SDPP in PS diets of weaned piglets, aiming to understand its gut barrier function related mode of action in improving performance, compared to control diet during the critical immediate post-weaning window, when the gut is highly vulnerable.

The inclusion of 5% SDPP in the PS diets of weaned piglets in this study significantly increased ADG, ADFI, and G:F during the first week post-weaning. These findings align with earlier studies demonstrating that SDPP stimulates feed intake probably due to its palatability [5,21], which is crucial during the weaning transition when pigs often exhibit reduced appetite [9]. Furthermore, the increment in both ADFI and G:F suggest that SDPP not only stimulated feed intake but also enhanced nutrient utilization efficiency, possibly due to its bioactive compounds. Understandably, even though the SDPP-fed piglets continued to perform better throughout the 35 d period trial, the effects were not significant; with the fact that SDPP was only provided in the PS phase (14 d) and therefore it is possible that the benefits may not be sustained without same continued dietary support. These findings are consistent with earlier studies summarized by Van Dijk et al [5], concluding that the beneficial effect of SDPP on ADG and ADFI is much more pronounced in the first week than in the second week after weaning. Interestingly, our results also align with those of Che et al [23] who also highlighted the short-term growth advantages of SDPP in weanling pig diets where 4% SDPP fed pigs had a significantly higher ADG and consequently a lower feed-to-gain ratio than those offered 4.88% soy protein concentrate, only from day 1 to 10 post-weaning. Similarly, in a meta-analysis, SDP increased ADG and ADFI by 36% and 17%, respectively, in the first week post-weaning (d 0–7), but the improvements were lower from d 7–14 (2% and 3%), d 0–14 (24% and 12%) and d 0–28 (6% and 4%) [10].

The short-lived performance effect of SDPP for one week after weaning observed in our study contrasts with previous findings which showed prolonged benefits into the second week or beyond [8]. Other studies have also suggested that the inclusion of SDP in weaning diets enhances gastrointestinal development during the critical first weeks post-weaning, with lasting positive effects on the animals’ resilience to stress later in life [24,25]. Boyer et al [24] demonstrated that dietary SDP supplementation during the first two weeks post-weaning influenced subsequent immunological responses and mitigated intestinal injury following a *Salmonella typhimurium* challenge. Similarly, Pujols et al [25] reported that pigs vaccinated against PCV-2, fed with SDPP for the first 14 d post-weaning, and later challenged with PCV-2 at d 63, exhibited heavier carcass weights at slaughter and reduced cumulative mortality throughout their lifespan. These findings highlight the potential of SDPP to provide long-term health and performance benefits when incorporated during the early post-weaning period. The discrepancies observed may be due to differences in SDPP inclusion, duration of offer, health status of animals, variations in diet composition, environmental conditions, or both, used in these studies.

Weaning, particularly due to dietary transition, is a crucial stressful stage in the life of a piglet that can result in intestinal barrier disorders, poor immunocompetence and ultimately poor growth performance [2]. Although we expected effects of SDPP on intestinal health and barrier function of piglets, in response to the weaning challenge, no effects on fecal consistency, serum levels of citrulline and calprotectin, or bile sIgA were observed in our study. This might be explained by a high health status of the piglets, because of the low-challenging experimental conditions of the study. The lower fecal scores observed for the control group support this. However, for studies in which the control piglets showed poorer scores, SDP resulted in significant improvements [26]. Peace et al [7] also reported that piglets receiving diets with 5% SDP exhibited reduced fecal scores on d 7 and 14 post-weaning and Bailey et al [27] showed that the inclusion of 6% SDP in an already low-protein diet decreased diarrhea incidence during the first 6 d post-weaning.

On the other hand, it has been previously demonstrated that weaning in piglets triggers an early but temporary change in the expression of inflammatory cytokine genes within the gut [28,29] as well as a prolonged and transient post-weaning intestinal inflammation response, lasting at least 15 d, characterized by the upregulation of IgA-producing cells and the gene expression of both pro- and anti-inflammatory cytokines, including *TNF-α*, *IFN-γ*, *IL-1α*, *IL-1β*, *IL-6*, *IL-8*, *IL-10*, *IL-12α*, and *TGF-β*, in the jejunum, ileum, and colon which subsequently can alter gut barrier function, metabolism, and growth [2,30,31].

Activation of the immune system increases nutrient utilization for immune response, thereby limiting nutrients for growth. Therefore, improving immune response efficiency by swiftly restoring homeostasis and maintaining intestinal barrier integrity can rapidly stabilize immune activation and redirect nutrients to productive functions. Dietary interventions, such as the use of SDPP in the current study, have demonstrated potential in modulating gene expression and regulating gut inflammatory processes [32], offering a dietary therapeutic approach to managing weaning-related stress. While dietary SDPP has been shown to mitigate these inflammatory effects [6], its precise immunomodulatory mechanisms remain only partially understood. The current study specifically investigated the influence of SDPP on the gene expression patterns in three intestinal sections (jejunum, ileum and cecum) of piglets after the first week post-weaning.

SDP offers a rich mixture of bioactive compounds that support the immune system, reducing the need for energy and nutrients to build and sustain an immune response and thereby mitigating the negative impacts of weaning stress [8]. The composition and bioactive compounds profile of SDPP have been previously reviewed by Torrallardona [8] and Kazimierska and Biel [33]. The mechanisms underlying the growth-promoting effects of SDPP in the face of weaning can be attributed to its rich composition of these functional proteins, including immunoglobulins, growth factors, and bioactive peptides. Diets that contain SDP are known to increase blood levels of IgG and IgA in pigs [23] and in comparison, to other protein sources records high number of bioactivities [34] thus playing important roles in growth and immunological support [22,34].

It has been previously noted that SDPP could modulate the expression of genes related to gut barrier function and immune response in nursery pigs [19] at d 14 post-weaning but data on this modulation remain limited. Therefore, our study offers novel insights into its immunomodulatory mechanisms of action just one week after weaning. In our study, the traversal gene expression analysis employing 45 genes of interest and covering a wide functional range revealed that only a limited number of genes specifically, *IL-1β*, *IL-8*, *GBP1*, and *TGF-β1* in the ileum and only *IL-1β* in jejunum were upregulated by the SDPP diet, suggesting a targeted immune response rather than a broad-spectrum effect.

Interleukin 1β is a proinflammatory cytokine which is considered a significant mediator of inflammation and tissue damage in inflammatory bowel disease [31,32], and is often released in response to injury, stress or microbial challenge [35]. Piglets fed SDP were previously shown to have slightly higher serum *IL-1β* levels than those not fed SDP [36]. Similarly, *IL8* is primarily expressed in enterocytes and macrophages to chemoattract other immune cells, particularly neutrophils, in response to an induced acute inflammatory process [37]. Our results showed that the expression of *NF-κB1* subunit was slightly upregulated by SDPP treatment (trend) suggesting IL-1β could participate in the *NF-κB* pathway activation, and the subsequent IL-8 release in ileum [38]. The upregulation of *IL8* and *IL-1β* by SDPP in the ileum is, therefore, indicative of an antigenic and immunomodulatory effect of SDPP that helps to recruit immune cells in absence of a real pathogenic challenge, promoting immune resilience against possible further infections.

The cytokine, *TGF-β1*, is a key regulator of intestinal adaptation in post-weaning piglets that is involved in promoting enterocyte proliferation, maintaining intestinal integrity, and modulating mucosal immune homeostasis [39,40]. The higher *TGF-β1* expression in SDPP pigs might be indicative of mucosal anti-inflammatory activity enabling the gut immune system to compensate for the *IL-1β* and *IL-8* induction by also enhancing counter-regulatory signals. Konkel and Chen [41] pointed to the dual role in anti-inflammatory activity and in epithelial repair, and that its expression is often context-dependent. Considering that *TGF-β* is also associated with the maintenance of intestinal integrity, it is likely that it reinforced the barrier function, an effect that would have been even higher under challenge conditions [6], especially given that SDP has been shown to modulate specific immune responses more strongly in early-weaned piglets and those under poor health conditions [42]. As an inflammatory regulator, *GBP-1* mediates the anti-proliferative effects of IFN-γ on epithelial cells while protecting the mucosa [43]. In the current trial, the SDPP upregulation of *GBP-1*, also reported by González-Solé et al [19], and *TGF-β1*, equally observed by Pérez-Bosque et al [44] further suggests that SDPP may play a role in modulating inflammatory responses and promoting tissue repair processes in the gut, as indicated by previous research that demonstrated the anti-inflammatory properties of dietary plasma [6].

While *IL-1β* and *IL-8* are proinflammatory proteins and *GBP-1* and *TGF-β1* are immunomodulatory genes, their targeted upregulation suggests that SDPP may help modulate the immune system through GALT regulation in a way that supports growth and intestinal health, thus providing a strategy of dealing with post-weaning challenges [45]. With these results we have established that diet supplementation with SDPP can reinforce the immune system, thereby regulating inflammatory responses to promote intestinal homeostasis and consequently improve growth performance in weaned piglets.

## CONCLUSION

This study validates the use of SDPP in the PS diets of weaned piglets, demonstrating significant benefits in gut health and performance. The observed improvements in feed intake, growth performance particularly in the first week and the targeted immunomodulation in the small intestine suggests that SDPP helps the gut adapt to the stressors associated with weaning. The selective upregulation of immune-related genes, including *IL-1β*, *IL-8*/*CXCL8*, *GBP-1*, and *TGF-β1*, highlights SDPP’s role in modulating inflammation, GALT regulation and promoting intestinal health. These findings support SDPP as a viable nutritional strategy for improving performance and gut health of weaned piglets. Future research should continue to explore these underlying mechanisms of action of SDPP, its effects both before and after weaning, and its influence on microbial diversity.

## Figures and Tables

**Table 1:** Ingredient and nutrient composition (as-fed basis) of diets

Item	Pre-starter (0–14 d)		Starter (15–35 d)
	
	Control	SDPP	
Ingredients (%)			
Barley	31.48	32.86	23.55
Corn	20.00	20.00	25.00
Wheat	-	-	20.00
Soybean meal (48% CP)	23.04	23.13	23.91
Sweet milk whey	6.50	6.50	-
Soy protein concentrate	6.20	-	-
SDPP	-	5.00	-
Dextrose	6.50	6.50	
Animal fat	2.94	3.17	3.76
L-Lys·HCl	0.29	0.18	0.50
L-Thr	0.14	0.06	0.22
DL-Met	0.18	0.12	0.18
L-Trp	0.04	0.01	0.05
L-Val	-	-	0.06
Salt	0.53	0.25	0.48
Calcium carbonate	0.03	-	0.24
Dicalcium phosphate	1.70	1.81	1.63
Noxyfeed^1)^	0.02	0.02	0.02
Vit-Min complex^2)^	0.40	0.40	0.40
Estimated nutrient content (%)			
Crude protein	20.50	20.50	18.00
Crude fibre	3.12	2.95	3.12
Fat	4.70	5.01	5.86
Ash	5.87	5.79	5.24
Metabolizable energy (kcal/kg)	3,300	3,300	3,300
Total calcium	0.750	0.754	0.750
Digestible phosphorous	0.380	0.380	0.330
SID Lys	1.250	1.250	1.200
SID Thr	0.813	0.813	0.780
SID Met	0.452	0.375	0.426
SID Met+Cys	0.738	0.740	0.708
SID Trp	0.250	0.250	0.240
SID Ile	0.796	0.758	0.674
SID Val	0.870	0.938	0.816
Analyzed nutrient content (%)			
Dry matter	89.7	89.3	89.5
Crude protein	20.7	20.5	17.6
Total fat	4.08	4.36	5.39
Ash	5.39	5.44	4.68
Gross energy (kcal/kg)	4,128	4,171	4,137

1) Noxyfeed 56P (ITPSA). It contains 56% of antioxidant substances (butylated hydroxytoluene+propyl gallate) and synergistic (citric acid 14%+authorized support).

2) Vitamin-Mineral premix (DEX Ibérica). Provided per kg feed: vitamin A (retinyl acetate) 10,000 IU; vitamin D_3_ (Cholecalciferol) 2,000 IU; vitamin E (all-rac-alpha-tocopheryl acetate) 25 mg; vitamin K_3_ (menadione sodium bisulfite) 1.5 mg; vitamin B_1_ (thiamine mononitrate) 1.5 mg; vitamin B_2_ (Riboflavin) 3.5 mg; vitamin B_6_ (pyridoxine hydrochloride) 2.4 mg; vitamin B_12_ 20 μg; nicotinic acid (niacin) 20 mg; calcium D-pantothenate 14 mg; folic acid 0.5 mg; biotin 50 μg; Fe (from FeSO_4_·H_2_O) 120 mg; I (from KI) 0.75 mg; Cu (from CuSO_4_·5H_2_O) 6 mg; Mn (from MnO) 60 mg; Zn (from ZnO) 65 mg; Se (E 8) (from Na_2_SeO_3_) 0.37 mg.

SDPP, spray-dried porcine plasma; CP, crude protein; Lys, lysine; Met, methionine; Val, valine; SID, standardized ileal digestible; Met+Cys, methionine+cysteine; Trp, tryptophan; Thr, threonine; Ile, isoleucine.

**Table 2:** Effects of SDPP supplementation to the control diet on the performance of weaned pigs

Item	Control	SDPP	SEM	p-value
BW (kg)				
0 d	8.18	8.15	0.450	0.248
7 d	9.11	9.59	0.473	0.014
14 d	11.32	11.71	0.553	0.207
35 d	20.86	21.38	0.829	0.482
ADG (g/d)				
0–7 d	133	205	12.4	<0.001
7–14 d	316	303	22.5	0.668
0–14 d	224	254	14.7	0.185
14–35 d	454	460	18.0	0.810
0–35 d	362	378	14.8	0.463
ADFI (g/d)				
0–7 d	215	280	13.6	0.009
7–14 d	468	466	25.9	0.960
0–14 d	341	373	18.2	0.219
14–35 d	724	697	30.6	0.470
0–35 d	571	568	23.8	0.898
G:F				
0–7 d	0.61	0.74	0.035	0.026
7–14 d	0.67	0.65	0.031	0.606
0–14 d	0.65	0.68	0.025	0.326
14–35 d	0.63	0.67	0.016	0.108
0–35 d	0.63	0.67	0.013	0.077

Values are presented as least squares means of 9 pens per treatment.

SDPP, spray-dried porcine plasma; SEM, standard error of the least square means; BW, body weight; ADG, average daily weight gain; ADFI, average daily feed intake; G:F, gain-to-feed ratio.

**Table 3:** Effects of SDPP on levels of calprotectin and citrulline in serum and sIgA in bile at 7 d post-weaning

Item	Control	SDPP	SEM	p-value
Calprotectin (ng/mL)	911	954	116.99	0.731
Citrulline (μmol/L)	53	63	6.72	0.161
Secretory IgA (ng/mL)	4,490	2,757	1,227.21	0.177

Values are presented as least squares means of 9 piglets per treatment.

SDPP, spray-dried porcine plasma; SEM, standard error of the least squares means.

**Table 4:** Effects of SDPP on intestinal mucosa gene expression at 7 d post-weaning

Function	Gene	Jejunum				Ileum				Caecum			
		
		Control	SDPP	SEM	p-value	Control	SDPP	SEM	p-value	Control	SDPP	SEM	p-value
BF	*OCLN*	1.03	0.92	0.125	0.343	1.71	2.31	0.334	0.242	0.83	0.91	0.118	0.367
	*CLDN15*	13.24	17.46	9.586	0.695	4.81	4.99	2.956	0.964	1.75	1.25	0.415	0.372
	*ZO1*	2.52	3.12	1.253	0.692	1.19	1.82	0.407	0.285	0.72	0.64	0.139	0.702
	*CLDN1*	25.65	29.27	16.986	0.838	9.22	10.82	6.020	0.841	5.83	2.23	2.622	0.273
	*MUC2*	1.18	1.41	0.247	0.468	0.80	0.77	0.128	0.852	0.87	1.13	0.163	0.101
	*MUC13*	0.51	0.52	0.101	0.976	0.55	0.59	0.122	0.856	0.23	0.36	0.072	0.226
	*CLDN4*	4.74	5.26	3.898	0.927	1.89	3.10	0.912	0.375	2.66	1.31	0.638	0.144
IR	*pBD3*	3.32	9.32	4.580	0.231	0.95	0.92	0.166	0.910	0.85	0.27	0.253	0.100
	*REG3G*	0.96	1.34	0.575	0.643	0.49	0.60	0.266	0.787	1.48	0.21	0.519	0.119
	*IL8/CXCL8*	1.90	2.35	0.682	0.614	0.58	1.12	0.154	0.010	1.87	1.81	0.440	0.926
	*IKBKB*	3.83	4.38	1.688	0.715	1.93	4.28	0.879	0.068	-	-	-	-
	*IL6*	0.97	0.98	0.333	0.979	0.55	0.55	0.112	0.984	1.23	1.33	0.255	0.997
	*TLR4*	1.53	1.06	0.296	0.290	0.63	0.83	0.118	0.274	0.75	0.79	0.205	0.917
	*pBD2*	0.57	0.83	0.174	0.305	0.91	1.15	0.178	0.252	0.64	0.98	0.156	0.117
	*GBP1*	1.20	1.23	0.257	0.936	0.92	1.62	0.194	0.014	0.82	0.72	0.110	0.495
	*IFNγ*	0.54	0.43	0.118	0.545	1.08	1.40	0.249	0.392	-	-	-	-
	*IL22*	0.56	1.09	0.298	0.247	0.19	2.53	1.019	0.148	1.23	1.29	0.463	0.971
	*TGF-β1*	1.38	1.48	0.222	0.746	1.04	1.59	0.127	0.015	1.26	1.18	0.196	0.661
	*TNFa*	2.23	3.49	1.392	0.361	0.79	1.08	0.167	0.246	1.86	2.05	0.396	0.769
	*IFNGR1*	0.72	0.70	0.103	0.826	0.95	1.12	0.149	0.401	0.68	0.72	0.184	0.865
	*TLR2*	1.63	2.88	0.842	0.334	0.69	1.36	0.227	0.072	0.72	0.94	0.145	0.124
	*NFKBIA*	3.90	3.89	1.909	0.984	1.70	2.10	0.635	0.619	0.00	0.00	0.000	0.288
	*DEFB1*	0.52	0.90	0.226	0.255	0.61	0.59	0.118	0.931	3.55	3.01	1.496	0.813
	*IL10*	1.98	1.42	0.431	0.294	0.71	1.00	0.120	0.121	1.74	0.85	0.415	0.068
	*IL17A*	2.51	2.28	0.768	0.834	0.30	1.36	0.591	0.234	0.52	0.73	0.246	0.567
	*CCL20*	1.85	2.51	0.768	0.516	0.39	0.63	0.162	0.279	1.87	1.20	0.460	0.317
	*NFκB1*	1.22	1.41	0.093	0.109	0.80	0.96	0.059	0.093	-	-	-	-
	*IL1β*	0.72	1.72	0.485	0.033	0.40	1.10	0.212	0.018	1.34	1.38	0.462	0.963
MEHA	*GPX2*	0.88	1.56	0.262	0.102	0.85	1.19	0.297	0.445	0.54	0.66	0.121	0.392
	*DAO*	0.63	1.24	0.383	0.288	1.77	2.74	0.735	0.379	0.12	0.35	0.091	0.116
	*PPARGC1A*	0.59	0.84	0.122	0.175	1.02	1.06	0.171	0.879	0.77	0.98	0.193	0.462
	*IGF1R*	3.02	3.34	1.361	0.859	1.03	1.20	0.129	0.315	0.60	0.96	0.145	0.103
	*IDO1*	0.42	1.73	0.727	0.134	0.60	0.97	0.177	0.180	1.54	0.94	0.400	0.142
	*FAXDC2*	1.35	1.81	0.443	0.342	3.64	3.11	1.180	0.760	1.09	0.79	0.316	0.710
	*SOD2*	1.26	1.28	0.152	0.960	1.12	1.35	0.218	0.455	0.74	0.76	0.139	0.868
	*HNMT*	1.03	1.14	0.131	0.540	1.46	1.75	0.172	0.266	1.09	1.07	0.246	0.959
	*CCK*	1.21	1.52	0.200	0.211	0.85	0.95	0.157	0.583	-	-	-	-
	*ALPI*	0.93	1.34	0.338	0.217	1.11	1.59	0.176	0.091	1.64	0.40	0.662	0.153
NT	*SLC5A1*	0.54	0.91	0.235	0.292	1.02	1.43	0.315	0.364	0.48	0.63	0.140	0.407
	*SLC7A8*	0.38	0.53	0.103	0.361	0.69	1.29	0.524	0.439	0.46	0.45	0.067	0.832
	*SLC16A1*	0.54	1.09	0.273	0.185	0.77	1.07	0.205	0.330	0.23	0.27	0.051	0.621
	*SLC39A4*	0.84	0.92	0.129	0.544	1.58	1.50	0.339	0.877	0.62	0.64	0.124	0.887
SI/IR	*HSPA4*	0.83	0.90	0.077	0.513	0.98	1.08	0.107	0.326	0.57	0.69	0.091	0.150
SI	*HSD11B1*	3.93	7.97	3.337	0.294	0.81	1.54	0.321	0.146	0.84	0.51	0.167	0.196

Values are presented as least squares means.

SDPP, spray-dried porcine plasma; SEM, standard error of the least square means.

Functions: BF, barrier function; IR, immune response; MEHA, metabolic, enzymatic and hormonal activity; NT, nutrient transport; SI, stress indicators.

Genes: *OCLN*, Occludin; *CLDN15*, Claudin-15; *ZO1*, Zonula occludens-1; *CLDN1*, Claudin-1; *MUC2*, Mucin 2; *MUC13*, Mucin 13; *CLDN4*, Claudin-4; *pBD3*, Porcine beta-defensin 3; *REG3G*, Regenerating-islet derived protein 3 gamma; *IL8/CXCL8*, Interleukin 8/C-X-C motif chemokine ligand 8; *IKBKB*, Inhibitor of nuclear factor kappa B kinase subunit beta; *IL6*, Interleukin 6; *TLR4*, Toll-like receptor 4; *pBD2*, Porcine beta-defensin 2; *GBP1*, Guanylate binding protein 1; *IFNγ*, Interferon gamma; *IL22*, Interleukin 22; *TGF-β1*, Transforming growth factor beta 1; *TNFα*, Tumor necrosis factor alpha; *IFNGR1*, Interferon gamma receptor 1; TLR2, Toll-like receptor 2; *NFKBIA*, NFκB inhibitor alpha; *DEFB1*, Porcine beta-defensin 1; *IL10*, Interleukin 10; *IL17A*, Interleukin 17A; *CCL20*, Chemokine (C-C motif) ligand 20; *NFκB1*, Nuclear factor kappa B subunit 1; *IL1β*, Interleukin 1 beta; *GPX2*, Glutathione peroxidase 2; *DAO*, D-amino acid oxidase; *PPARGC1A*, Peroxisome proliferative activated receptor gamma; *IGF1R*, Insulin-like growth factor 1 receptor; *IDO1*, Indoleamine 2,3-dioxygenase; *FAXDC2*, Fatty acid hydrolase domain containing 2; *SOD2*, Superoxide dismutase; *HNMT*, Histamine N-methyltransferase; *CCK*, Cholecystokinin; coactivator 1 alpha; *ALPI*, Intestinal alkaline phosphatase; *SLC5A1*, Solute carrier family 5 (sodium/glucose cotransporter) member 1; *SLC7A8*, Solute carrier family 7 (amino acid transporter light chain, L System) member 8; *SLC16A1*, Monocarboxylate transporter 1; *SLC39A4*, Solute carrier family 39 (zinc transporter) member 4; *HSPA4*, Heat shock protein 70; *HSD11B1*, Hydroxysteroid (11-beta) dehydrogenase 1.

## SUPPLEMENTARY MATERIAL

**Supplement 1**. List and details of primers used in the high-throughput quantitative real-time PCR.
