# Supplementary material for: Spray-dried porcine plasma improves piglets’ performance and modulates gut immune-related genes in the first week post-weaning
Source: Anim Biosci. 2025 Jun 10;38(11):2475–86. doi: 10.5713/ab.25.0185 (PMC12580963; doi:10.5713/ab.25.0185)
Supplement: Supplementary file 1 [file ab-25-0185-supplementary-1.pdf]

Supplement 1. List and details of primers used in the high-throughput quantitative real-time PCR.

Function	*Gene*	*Name*	*5'->3'*	*Primer Sequence*	*Accession number*	*Tm*	*GC%*	*Amplicon length*
Permeability and Intestinal Barrier	*OCLN*	Occludin	Forward	CAGGTGCACCCTCCAGATTG	NM_001163647.2	60.68	60.00	126
			Reverse	AGGCCTATAAGGAGGTGGACTT		60.02	50.00	
	ZO1	Zonula occludens-1	Forward	GGCTATGTCCAGAATCTCGGAAAA	XM_021098856.1	60.68	45.83	143
			Reverse	TGCTTCTTTCAATGCTCCATACC		59.30	43.48	
	CLDN1	Claudin-1	Forward	ACCCCAGTCAATGCCAGATATG	NM_001244539.1	60.16	50.00	91
			Reverse	AAAGTAGGGCACCTCCCAGAAG		61.69	54.55	
	CLDN4	Claudin-4	Forward	CCTCCGTGCTGTTCCTCAA	XM_005661969.2	59.63	57.89	83
			Reverse	GAGGCACAAGCCCAGCAA		60.60	61.11	
	CLDN15	Claudin-15	Forward	GGATGGTGGCTATCTCCTGGTA	NM_001161643.1	60.76	54.55	88
			Reverse	GGGCCCAGCTCATACTTGGT		61.93	60.00	
	MUC2	Mucin 2	Forward	GGACGACACCATCTACCTCACT	XM_021082584.1	61.21	54.55	131
			Reverse	GGCCAGCTCGGGAATAGAC		59.93	63.16	
	MUC13	Mucin 13	Forward	CAGTGGAGTTGGCTGTGAAAAC	NM_001105293.1	60.22	50.00	150
			Reverse	ATCAAGTTCTGTTCTTCCACATTCTTG		60.26	37.04	
Oxidative state	*GPX2*	Glutathione peroxidase 2	Forward	GCAACCAATTTGGACATCAGGAG	NM_001115136.1	60.37	47.83	99
			Reverse	GGGTAAAGGTGGGCTGGAAT		59.66	55.00	
	SOD2	Superoxide dismutase	Forward	GGGGTTGGCTCGGTTTCAA	NM_214127.2	60.53	57.89	123
			Reverse	CATGCTCCCACACGTCGAT		60.15	57.89	
Digestion and Metabolism	*ALPI*	Intestinal alkaline phosphatase	Forward	ATGTCTTCTCTTTTGGTGGCTACA	XM_003133729.4	60.20	41.67	92
			Reverse	GGAGGTATATGGCTTGAGATCCA		59.16	47.83	
	DAO	D-amino acid oxidase	Forward	GAACCAACAGACCTTCAACTATCTC	NM_214066.2	59.36	44.00	148
			Reverse	CTTTCGGAATCCCAGGACCAT		59.79	52.38	
	HNMT	Histamine N-methyltransferase	Forward	TGTTGAACCAAGTGCTGAACAAAT	NM_001244561.1	60.08	37.50	76
			Reverse	CTTTATGTTCTCGAGGTTTGATGTCTT		59.88	37.04	
	IDO1	Indoleamine 2,3 dioxygenase	Forward	GTCTTGGCAAATTGGAAGAAAAAGG	NM_001246240.1	59.76	40.00	80

			Reverse	CCCGGAAATGAGAAGAGAATATCCAT		60.52	42.31	
	CCK	Cholecystokinin	Forward	CAGCAGGCTCGAAAAGCAC	NM_214237.2	59.79	57.89	109
			Reverse	AATCCATCCAGCCCATGTAGTC		59.89	50.00	
	IGF1R	Insulin-like growth factor 1 receptor	Forward	CCGACGCGGCAACAAC	NM_214172.1	59.40	68.75	116
			Reverse	TCAGGAAGGACAAGGAGACCAA		60.70	50.00	
	PPARGC1A	Peroxisome proliferative activated receptor gamma, coactivator 1 alpha	Forward	CTCTGGAACTGCAGGCCTAA	NM_213963.2	59.38	55.00	79
			Reverse	TGGAGAAGCCCTAAAAGGGTTAT		59.14	43.48	
Immune Response	*IL1β*	Interleukin 1 beta	Forward	GGTGACAACAATAATGACCTGTTATTTG	NM_214055.1	59.71	35.71	99
			Reverse	GCTCCCATTTCTCAGAGAACCA		60.03	50.00	
	IL6	Interleukin 6	Forward	TCCAATCTGGGTTCAATCAGGAG	NM_214399.1	60.05	47.83	124
			Reverse	ACAGCCTCGACATTTCCCTTATT		60.05	43.48	
	IL8/CXCL8	Interleukin 8/C-X-C motif chemokine ligand 8	Forward	GGAAAAGTGGGTGCAGAAGGT	NM_213867.1	60.75	52.38	96
			Reverse	GGAGAATGGGTTTTTGCTTGTTGT		60.68	41.67	
	IL10	Interleukin 10	Forward	GAGGCTGCGGCGCT	NM_214041.1	59.30	78.57	84
			Reverse	AGCTTGCTAAAGGCACTCTTCA		60.22	45.45	
	IL22	Interleukin 22	Forward	TGTTCCCCAACTCTGATAGATTCC	XM_021091968.1	59.84	45.83	131
			Reverse	GTTGTTCACATTTCTCTGGATATGCT		59.91	38.46	
	IL17A	Interleukin 17A	Forward	CCAGACGGCCCTCAGATTAC	NM_001005729.1	59.89	60.00	144
			Reverse	GATCTTCCTTCCCTTCAGCATTG		59.37	47.83	
	TLR2	Toll-like receptor 2	Forward	CTCTCGTTGCGGCTTCCA	NM_213761.1	60.05	61.11	115
			Reverse	AAGACCCATGCTGTCCACAAA		60.13	47.62	
	TLR4	Toll-like receptor 4	Forward	CATCCCCACATCAGTCAAGATACT	NM_001113039.2	59.90	45.83	130
			Reverse	GTCAATTGTCTGAATTTCACATCTGG		59.19	38.46	
	TNFα	Tumor necrosis factor alpha	Forward	ACCACGCTCTTCTGCCTACT	NM_214022.1	60.90	55.00	132
			Reverse	GACGGGCTTATCTGAGGTTTGA		60.09	50.00	
	IFNγ	Interferon gamma	Forward	AAGAATTGGAAAGAGGAGAGTGACA	NM_213948.1	59.93	40.00	101

			Reverse	TGAATGGCCTGGTTATCTTTGA		57.69	40.91	
	IFNGR1	Interferon gamma receptor 1	Forward	CATGTTACCCAAATCTTTGCTGTCT	NM_001177907.1	60.05	40.00	149
			Reverse	CAGTATGCACGCTTGAAATTGTC		59.15	43.48	
	HSPA4	Heat shock protein 70	Forward	TCTCAATTGCCTGCGATTAATGAA	XM_005661654.3	59.36	37.50	127
			Reverse	AGAATGCCCCATGTCTACAAAAAC		59.78	41.67	
	CCL20	Chemokine (C-C motif) ligand 20	Forward	AGACCATATTCTTCACCCCAGATTT	NM_001024589.1	59.81	40.00	113
			Reverse	CACACACGGCTAACTTTTTCTTTG		59.50	41.67	
	REG3G	Regenerating-islet derived protein 3 gamma	Forward	TGCCTGATGCTCCTGTCTCA	NM_001144847.1	60.91	55.00	111
			Reverse	GGCATAGCAGTAGGAAGCATAGG		60.62	52.17	
	FAXDC2	Fatty acid hydrolase domain containing 2	Forward	CCATGACTACCACCATCTCAAGTT	XM_005672602.2	60.32	45.83	121
			Reverse	CAGGATCGTGTGTCTCTCGTA		58.99	52.38	
	GBP1	Guanylate binding protein 1	Forward	GAATCCATCACAGCAGACGAGTA	NM_001128473.1	60.18	47.83	99
			Reverse	GATACAGAGTCGAGGCAGGTTAA		59.62	47.83	
	DEFB1	Porcine beta-defensin 1	Forward	GTATTCCTCCTCATGGTCCTGTT	NM_213838.1	59.54	47.83	134
			Reverse	CAGGTGCCGATCTGTTTCATC		59.33	52.38	
	pBD2	Porcine beta-defensin 2	Forward	ACTGTCTGCCTCCTCTCTTCC	NM_214442.2	60.89	57.14	154
			Reverse	TGTAACAGGTCCCTTCAATCCTG		59.99	47.83	
	pBD3	Porcine-beta-defensin 3	Forward	ACCTTCTCTTTGCCTTGCTCTT	NM_214444.1	60.16	45.45	164
			Reverse	GCCACTCACAGAACAGCTACC		60.94	57.14	
	IKBKB	Inhibitor of nuclear factor kappa B kinase subunit beta	Forward	TGGGATCACATCGGACAAACTG	NM_001099935.1	60.62	50.00	85
			Reverse	CTTCACCTCGTTCTCCCGTC		60.11	60.00	
	NFκB1	Nuclear factor kappa B subunit 1	Forward	TCCACAAGGCAGCAAATAGA	NM_001048232.1	57.12	45.00	83
			Reverse	AAGCTGAGTTTGCGAAAGGA		58.03	45.00	
	NFKBIA	NFκB inhibitor alpha	Forward	GAGGATGAGCTGCCCTATGAC	NM_001005150.1	60.00	57.14	85
			Reverse	CCATGGTCTTTTAGACACTTTCC		57.37	43.48	
	TGF-β1	Transforming growth factor beta 1	Forward	CTGGCCCCCAGTGACTCA	NM_214015.2	60.93	66.67	96
			Reverse	GCGAAAACCCTCTATAGCCTCTCT		61.77	50.00	

Nutrient transporters	*SLC5A1*	Solute carrier family 5 (sodium/glucose cotransporter) member 1	Forward	GGCCATCTTTCTCTTACTGGCA	NM_001164021.1	60.36	50.00	147
			Reverse	CCTCCCACTTCATGAAAAGCAAAC		60.80	45.83	
	SLC7A8	Solute carrier family 7 (amino acid transporter light chain, L System) member 8	Forward	GTCGCTTATGTCACTGCAATGT	XM_021099239.1	59.58	45.45	122
			Reverse	GACAGGGCGACGGAAATG		58.51	61.11	
	SLC16A1	Monocarboxylate transporter 1	Forward	CCTTGTTGGACCTCAGAGATTCTC	NM_001128445.1	60.62	50.00	132
			Reverse	CAGTATGTGTATTTATAGTCTCCGTATATGTC		59.69	34.38	
	SLC39A4	Solute carrier family 39 (zinc transporter) member 4	Forward	ATCTTTGGGCTCTTGCTCCTT	XM_001925360.5	59.64	47.62	139
			Reverse	GCAGCCCCAGCACCTTAG		60.44	66.67	
Stress	*HSD11B1*	Hydroxysteroid (11-beta) dehydrogenase 1	Forward	GTCAGAAGAAACTCTCAAGAAGGTG	NM_214248.3	59.53	44.00	99
			Reverse	GCGAAGGTCATGTCCTCCAT		59.82	55.00	
Reference genes	*GAPDH*	Glyceraldehyde-3-phosphate dehydrogenase	Forward	TTCGTCAAGCTCATTTCCTGGTA	NM_001206359.1	59.99	43.48	129
			Reverse	CCTCGCGTGCTCTTGCT		60.09	64.71	
	ACTB	Beta-actin	Forward	AAGGACCTCTACGCCAACAC	XM_021086047.1	59.68	55.00	130
			Reverse	CTGGAGGCGCGATGATCTT		59.93	57.89	
	TBP	TATA-box binding protein	Forward	ACAGAATGATCAAACCGAGAATTGT	XM_021085497.1	59.29	36.00	80
			Reverse	TGCTCTGACTTTAGCACCTGTTAA		60.20	41.67	
	HPRT1	Hypoxanthine phosphoribosyltransferase 1	Forward	TCATTATGCCGAGGATTTGGA	NM_001032376.2	57.14	42.86	91
			Reverse	CTCTTTCATCACATCTCGAGCAA		58.82	43.48	

TM – Melting Temperature, GC% - percentage of guanine and cytosine nucleotides in the primer sequence
